# Comparative study of sub-second temporal resolution 4D-MRI and 4D-CT for target motion assessment in a phantom model

**DOI:** 10.1038/s41598-023-42773-z

**Published:** 2023-09-21

**Authors:** Tianyuan Wang, Keitaro Sofue, Ryuji Shimada, Takeaki Ishihara, Ryuichi Yada, Masanori Miyamoto, Ryohei Sasaki, Takamichi Murakami

**Affiliations:** 1 Department of Radiation Oncology, Kobe University Hospital, Kobe, Japan; 2 Department of Radiology, Kobe University Graduate School of Medicine, 7-5-2, Kusunoki-cho, Chuo-ku, Kobe, 650-0017 Japan; 3 Center for Radiology and Radiation Oncology, Kobe University Hospital, Kobe, Japan

**Keywords:** Radiotherapy, Magnetic resonance imaging

## Abstract

To develop and investigate the feasibility of sub-second temporal resolution volumetric T1-weighted four-dimensional (4D-) MRI in comparison with 4D-CT for respiratory-correlated motion assessment using an MRI/CT-compatible phantom. Sub-second high temporal resolution (0.5 s) gradient-echo T1-weighted 4D-MRI was developed using a volumetric acquisition scheme with compressed sensing. An MRI/CT-compatible motion phantom (simulated liver tumor) with three sinusoidal movements of amplitudes and two respiratory patterns was introduced and imaged with 4D-MRI and 4D-CT to investigate the geometric accuracy of the target movement. The geometric accuracy, including centroid position, volume, similarity index of dice similarity coefficient (DSC), and Hausdorff distance (HD), was systematically evaluated. Proposed 4D-MRI achieved a similar geometric accuracy compared with 4D-CT regarding the centroid position, volume, and similarity index. The observed position differences of the absolute average centroid were within 0.08 cm in 4D-MRI and 0.03 cm in 4D-CT, less than the 1-pixel resolution for each modality. The observed volume difference in 4D-MRI/4D-CT was within 0.73 cm^3^ (4.5%)/0.29 cm^3^ (2.1%) for a large target and 0.06 cm^3^ (11.3%)/0.04 cm^3^ (11.6%) for a small target. The observed DSC values for 4D-MRI/4D-CT were at least 0.93/0.95 for the large target and 0.83/0.84 for the small target. The maximum HD values were 0.25 cm/0.31 cm for the large target and 0.21 cm/0.15 cm for the small target. Although 4D-CT potentially exhibit superior numerical accuracy in phantom studies, the proposed high temporal resolution 4D-MRI demonstrates sub-millimetre geometric accuracy comparable to that of 4D-CT. These findings suggest that the 4D-MRI technique is a viable option for characterizing motion and generating phase-dependent internal target volumes within the realm of radiotherapy.

## Introduction

Four-dimensional CT (4D-CT) is essential for determining respiration-related target motion while avoiding tumour under-dosage and explaining in detail organ at risk toxicity when adopted for radiotherapy planning^1^. Generally, an internal target volume derived using 4D-CT is used to assess respiratory motion and determine treatment margins for a moving target^2^. However, for a liver tumour, the poor soft-tissue contrast in conventional 4D-CT does not provide sufficient information to determine the target or assess its motion. Contrast-enhanced CT is generally used in clinical practice to obtain additional information^3^. However, a 4D-CT scan is usually inappropriate because acquiring the target region requires several minutes (mostly the entire liver). In addition, the axial direction of CT scan introduces undesirable motion artefacts, particularly in the superior-inferior direction, during high respiratory rate or irregular respiration^4^.

Respiratory-correlated 4D-MRI is a promising modality for evaluating the target motion of liver cancer in radiotherapy because of its high soft-tissue contrast and longer visualization capturing target motion without radiation exposure. This approach also provides a non-axial scanning direction, more desirable with fewer motion artefacts in the superior-inferior direction. Compared with 2D multi-slice acquisition-based MRIs^5,6^, 3D or volumetric acquisition-based 4D-MRIs have exhibited better geometric corrections, motion averaging, and image acceleration options^7–9^. However, previously reported volumetric 4D-MRI presented limitations regarding gaining sufficient temporal-spatial resolution while maintaining the image quality for tumour-to-tissue contrast^7,10,11^. Moreover, previous studies primarily applied single-shot T2-weighted or balanced steady-state free-precession sequences, where boundaries between the tumour and background liver tissue cannot be clearly defined^12^.

In order to address the limitations of poor soft tissue contrast in the liver site when determining moving targets using 4D-CT, as well as the challenges in achieving sufficient temporal-spatial resolution in previously reported 4D-MRI techniques, we have developed a 4D-MRI methodology based on gradient-echo 3D T1-weighted images (hepatobiliary phase) that attains high temporal-spatial resolution while maintaining optimal tumor-to-liver contrast.The gradient-echo 3D T1-weighted fast imaging is one of the basic sequences in liver MRI^13^. This sequence is used for hepatobiliary phase images that are acquired approximately 20 min after administration of gadoxetic acid contrast agent. The hepatobiliary phase image shows the highest tumour-to-liver contrast, lesion delineation, and continuously contrast enhancement in several hours^14^. In proposed 4D-MRI sequence, recently developed compressed sensing, enabling accelerated MRI acquisitions using the reconstruction of sparse images from highly under-sampled data^15,16^, was implemented to achieve sub-second temporal resolution. To the best of our knowledge, there are only a limited number of studies^17,18^ that have evaluated the T1-weighted 4D-MRI sequence in the existing literature. The proposed 4D-MRI method adopts a distinct approach from those previously reported. The aim of the present study is twofold: firstly, to employ the proposed 4D-MRI methodology to achieve sub-second temporal resolution in conjunction with high spatial resolution and image contrast; and secondly, to assess its accuracy by evaluating respiratory-correlated motion and comparing it with 4D-CT.

## Materials and methods

This study is Institutional Review Board exempt from the Kobe University Hospital as no actual patient data was used. All methods were carried out in accordance with relevant guidelines and regulations in our institution.

### Phantom preparation

#### Phantom geometry

**Figure 1:**
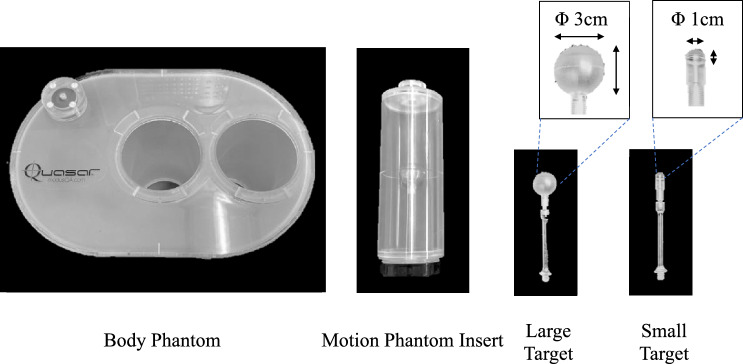
A QUASA MRI4D motion phantom body (left), motion phantom insert (middle), and two sizes of the simulated tumour (right) that can be set inside the motion phantom insert.

A commercial MRI/CT-compatible motion phantom (QUASA MRI4D Motion Phantom, ModusQA Medical Device), which measures 20 × 30 cm in diameter and 20 cm in length, was employed to conduct the evaluation systematically. The default phantom insert included a 3-cm-diameter spherical object as a simulated tumour. Additionally, a 1-cm-diameter insert to simulate a small-size tumour was also fabricated in this study (Fig. 1). External surrogate signals were used in both modalities for consistent comparison; a bellow (an airbag with a pressure sensor) was used for 4D-MRI, and an infrared reflective cube sensor for 4D-CT. The performances of several respiratory patterns in terms of different respiratory periods and motion amplitudes were quantified using two shapes of the simulated target.

#### Contrast agent solution

The contrast agent gadolinium-ethoxybenzyl-diethylenetriamine-pentaacetic-acid (Gd-EOB-DTPA, SH L 569 B, Primovist^®^) was used to prepare the optimal solution inside the phantom to gain target-to-tissue contrast similar to the target and normal tissue in gradient-echo 3D-T1-TFE (hepatobiliary phase image for liver cancer). Target-to-tissue in 4D-CT was necessary for comparison purposes using the same phantom settings. Hence, a contrast medium solution was prepared using 1000 mL of distilled water, 2 mL of Gd-EOB-DTPA (EOB Primovist^®^ Inj Syringe, BAYER), and 12 mL of Iohexol (OMNIPAQUE^®^ 300 inject syringe, GE healthcare) to simulate the hepatobiliary phase image of the liver in MRI and equilibrium phase image in CT (the solution was referred to in a previous study^19^ and modified). For a 3-cm-diameter target in MRI, the simulated tumour was filled with distilled water and its exterior with a contrast agent solution. In clinical practice, the simulated tumour is present at a lower signal without EOB, unlike a normal liver, which is present at a higher signal. For a 1-cm-diameter target in the MRI, the simulated tumour was injected with the EOB/Iohexol contrast agent solution and its exterior with water because creating a spherical object with a 1-cm-diameter was challenging. A possible workaround could be creating a 1-cm-diameter cavity inside a cylindrical stick to simulate the small-sized simulated tumour. In this case, only the spherical cavity would be present at a higher signal.

#### Motion pattern

The sinusoidal movements of amplitudes 1, 2, and 3 cm along the longitudinal axis were used in two respiratory settings: a regular respiratory pattern with 5 s/cycle and a fast respiratory pattern with 3 s/cycle. The sinusoidal motion patterns were modelled as the fourth power of a sinusoidal wave function, as reported in^20^.

$$y=A{sin}^{4}\left(2{\uppi}\frac{t}{2T}\right)-\frac{A}{2},$$

where *A* and *T* represent the amplitude of the motion and respiratory cycle, respectively.

### 4D-MRI image acquisition

**Figure 2:**
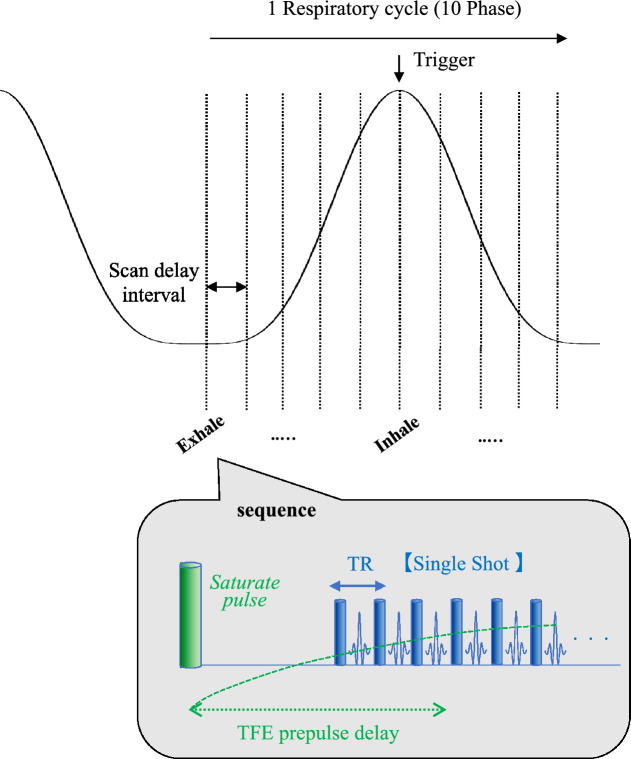
Acquisition process of the volumetric MRI data. The trigger signal was set at the exhale phase, and the delay interval for acquiring the next phase was specifically set for each respiratory pattern to acquire 10-phase volumetric data. The details of the scan sequence are shown below.

**Figure 3:**
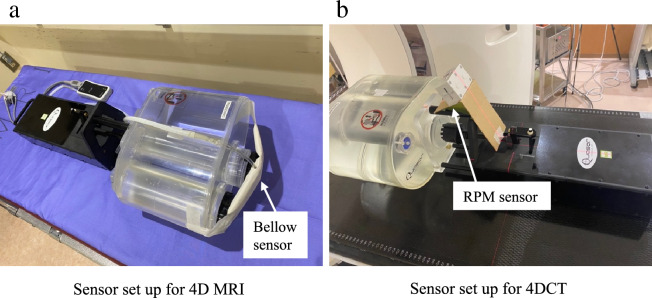
(**a**) Sensor setup for 4D-MRI (left) and 4D-CT (right). For 4D-MRI, the bellow sensor was set to the position where it could stably detect the movement from the motion phantom insert. (**b**) For 4D-CT, the RPM sensor was set to the position where it could stably detect the phantom motion. RPM refers to real-time position management.

To achieve sub-second high temporal resolution, our developed sequence features a single-shot 3D acquisition technique, which incorporates both half-scan and compressed sensing techniques. The 4D-MRI acquisition process is presented in Fig. 2. However, a potential limitation of this approach is the reduction in image contrast resulting from an increased TFE (Turbo Field Echo) factor. Consequently, the use of contrast agent in 4D-MRI was motivated by the desire to attain high temporal resolution imaging without compromising image contrast. Volumetric (3D) MRI data were acquired using a clinical MRI 3 T scanner (Ingenia ver. 5.4, Philips Medical Systems) with the following parameters: 3D-T1-TFE; TFE prepulse: saturate; TFE shot duration: 832 ms (849 ms) for a large (small) target; FOV 256 × 256 mm^2^; temporal resolution: 0.5 s; spatial resolution: 1.45 × 1.45 × 3 mm^3^ with 15 slices (1.45 × 1.14 × 2 mm^3^ with 12 slices) for a large (small) target; flip angle: 5°; TR/TE = 2.5/1.26 ms (2.6/1.25 ms) for a large (small) target; readout bandwidth: 1393 Hz/pixel; no fat suppression; compressed SENSE (Philips Medical Systems); factor: 5; and de-noising: strong. The half Fourier factor values utilized in this study were Y = 0.7 and Z = 0.8. The TFE (Turbo Field Echo) factor was set to 204, while the gradient mode was adjusted to its maximum setting. In this study, we employed a commercial application of the compressed sensing technique (Compressed SENSE). This approach integrates Compressed Sensing^15,16^ and SENSE technologies, and allows for user-defined reduction rates and denoising levels. When applied to 3D data acquisition, Compressed SENSE minimizes motion artifacts by setting a high reduction rate, which maintains image quality while significantly reducing acquisition time. To acquire 10-phase volumetric data based on each respiratory cycle, we used an external bellow sensor (an airbag with a pressure sensor) to monitor the respiratory cycles and acquire data using the triggered signal set at the inhale phase. Figure 3a illustrates the setup for the bellow sensor, configured to stably detect movement using the phantom insert. The delay interval for acquiring the next phase was specifically set for each respiratory pattern (e.g., 500 ms for 5 s/cycle) to acquire the 10-phase volumetric data (thrice). The 10-phase image data were sequentially binned based on the respiratory profile to generate the 4D image series. A static image series without motion was used as a reference.

### 4D-CT image acquisition

The 4D-CT images were acquired using the clinical multi-slice (16 detector rows) CT scanner (Aquilion LB, Canon Medical Systems). For CT scan protocol, the following clinical exposure settings were used: 120 kVp; 100 mA (200 mA) for a large (small) target; FOV: 500 × 500 mm^2^; gantry rotation speed: 0.5 s; and spatial resolution: 0.98 × 0.98 × 2 mm^3^. The respiratory phase was detected using the Varian real-time position management (RPM) system (Varian Medical Systems, Inc.) with an infrared reflective cube sensor positioned as in Fig. 3b. Similarly, a static image series without motion was used as a reference.

### Image analysis and statistical analyses

All the images were transferred to the MIM Maestro version 6.9.6 (MIM Software Inc, Cleveland, OH, USA) for target segmentation. To minimize the potential bias arising from the differing resolutions of the two modalities, we resampled images for both modalities to a resolution of 1 × 1 × 1 mm^3^ prior to performing ROI generation. This ensured that the same resolution was employed for delineating ROIs in both modalities. The reference target was contoured based on the static image series. A region growth method was used for objective and repeatable segmentation of each image series. The upper and lower thresholds of region growth were determined by generating the reference target region of interest (ROI). Minimal manual modification was used when the method did not perform properly.

#### Position accuracy evaluation

The target position was determined from the centroid of the target ROI for each image set. The exhale phase was normalised for reference, and the displacement distances of other phases were calculated with respect to it. The target and ground truth (GT) positions derived from the sinusoidal model were plotted and compared to evaluate their accuracy.

#### Target volume and similarity index evaluation

We utilized static images as a reference for each modality to assess volume and shape variations of the imaged object due to motion artifacts. The rationale behind using static images as a reference for each modality was the difficulty in discerning the plastic wall of the target phantom insert (as illustrated in Fig. 1) and the stem using MRI. Consequently, we defined the reference ground truth volume in the static image for each modality; specifically, for 4D-MRI/4D-CT, the large and small target volumes were 16.25 cc/14.13 cc and 0.57 cc/0.33 cc, respectively. The volume difference between the measurements and the reference GT was calculated as:

$$ {\text{dV }}\%  = \left( {{\text{V}}_{{{\text{measured}}}} {-}{\text{ V}}_{{{\text{ref}}}} } \right){\text{/V}}_{{{\text{ref}}}} \times { 1}00 \, \% . $$

where V_measured_ and V_ref_ represent the measured and actual volumes of the spherical object, respectively.

The volume difference could not reveal the extent of the shape difference between ROIs. Hence, the similarity index of each respiratory phase was evaluated using the Dice similarity coefficient (DSC)^21^ and Hausdorff distance (HD)^22^. The ROI of each phase was shifted with a constant displacement (based on the centroid position) in the superior-inferior direction with respect to the reference position. Moreover, the similarity index between the shifted and reference ROIs was calculated. The Mann–Whitney U-test was used to compare the performances of the two modalities (n = 60). Statistical calculations were performed in MATLAB 2022a (MathWorks, Natick, MA, USA). For statistical analyses, the reported *p* values were two-tailed; a *p* value < 0.05 was considered significant. A flowchart was included in Supplementary Material 2 to illustrate the methodology employed in this study.

## Results

**Figure 4:**
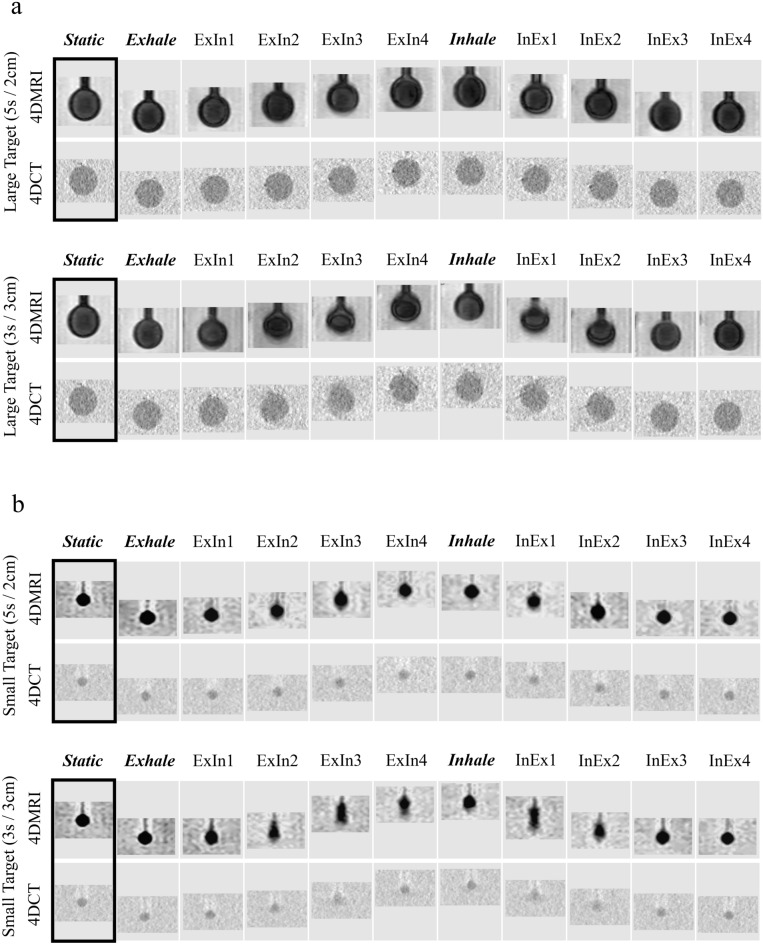
Acquired 4D-MRI and 4D-CT images with ten respiratory phases in a regular motion pattern (5 s/2 cm) and fast motion pattern (3 s/3 cm). The upper and lower panels show the results of large and small targets, respectively. The static image as a reference is shown on the left. ExIn refers to the phase from exhaling to inhaling, and InEx refers to the phase from inhaling to exhaling.

Figure 4 shows the acquired 4D-MRI and 4D-CT images with ten respiratory phases in a regular (5 s/2 cm) and fast (3 s/3 cm) motion pattern. The static image for each modality is presented on the left of the figure. In a regular motion pattern for large targets, 4D-MRI and 4D-CT represent visually comparable target-to-tissue contrasts with a sharp boundary in each respiratory phase. In a fast-motion pattern for large targets, a fast-moving phase (e.g., ExIn3, InEx1) revealed a slightly deformed target shape and blurred boundary in both modalities. For small targets in 4D-MRI, the fast-moving phase showed a large deformed shape along the movement direction while maintaining the target-to-tissue contrast with a sharp boundary. Contrarily, the target-to-tissue contrast for 4D-CT decreased in the fast-moving phase and the blurred target boundary (e.g., ExIn2-3, InEx1-2). Supplementary Material 1 presents a zoomed-out view of the imaged phantom with as much detail as possible.

### Position accuracy evaluation

**Figures 5:**
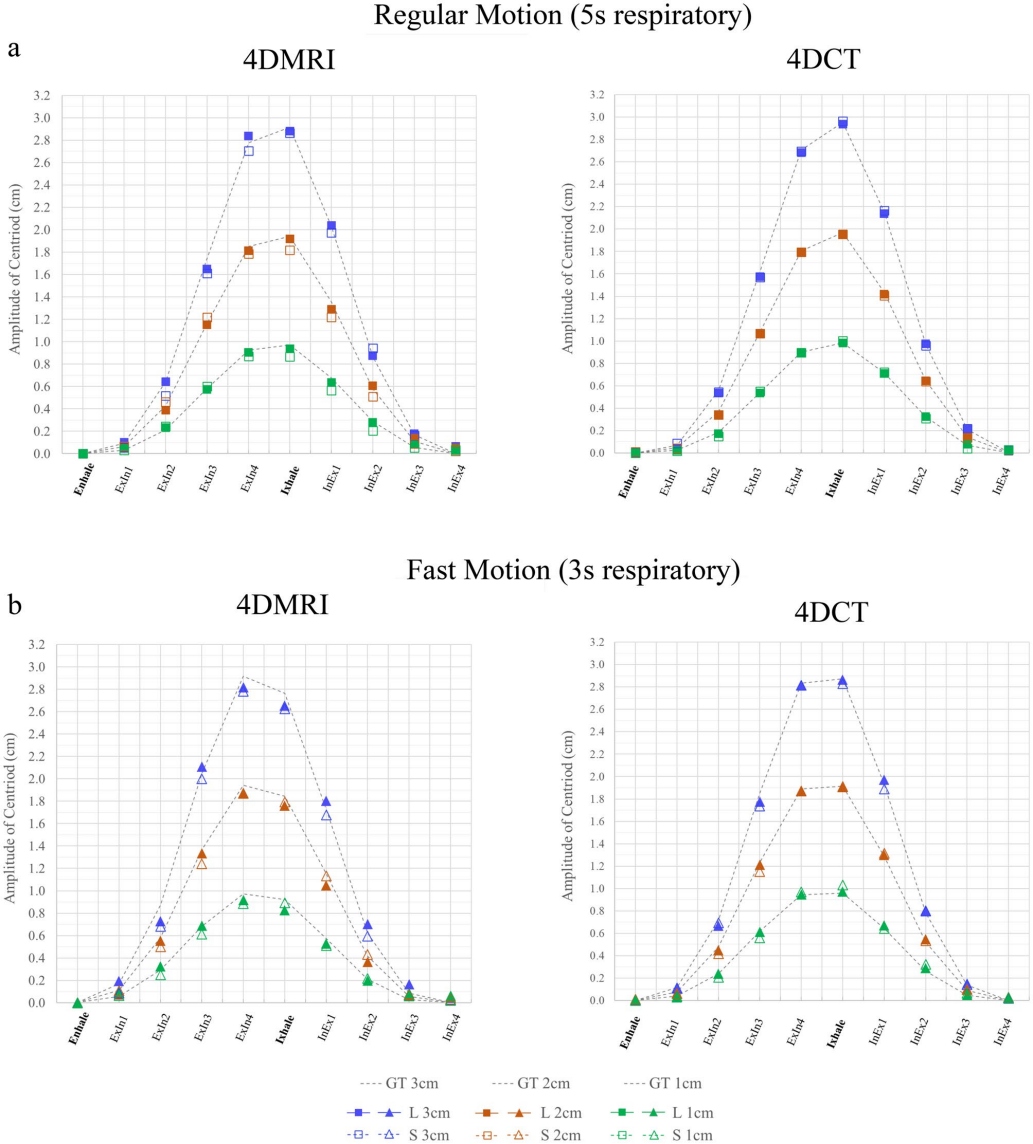
Position accuracy for each imaging modality with ten respiratory phases in (**a**) regular motion pattern (5 s respiratory) and (**b**) fast motion pattern (3 s respiratory). The results for the ground truth (dashed line), large target (solid), and small target (hollow) are plotted in the same figure. ExIn refers to the phase from exhaling to inhaling, and InEx refers to the phase from inhaling to exhaling. GT refers to ground truth. L and S refer to large and small targets, respectively.

The results of position accuracy analysis are plotted in Fig. 5. The averaged position difference and standard deviation from GT are summarised in Table 1. For a regular motion pattern, the absolute averaged position difference was observed within 0.06 and 0.02 cm for 4D-MRI and 4D-CT, respectively. Moreover, for the fast-motion pattern, the absolute averaged position difference was observed within 0.08 and 0.03 cm for 4D-MRI and 4D-CT, respectively. For both image modalities, the position accuracy of the small target was comparable to that of the large target. Although the average difference (0.05 cm) was less than 1-pixel resolution in both modalities, the results of the Mann–Whitney test suggested that 4D-CT outperformed the position accuracy of 4D-MRI (*p* < 0.01 for both large and small targets) in each case.

**Table 1:** Averaged absolute target position difference (cm).

		Amplitude 1 cm		Amplitude 2 cm		Amplitude 3 cm	
		4DMRI	4DCT	4DMRI	4DCT	4DMRI	4DCT
Large	Regular	0.02 ± 0.01	0.01 ± 0.01	0.03 ± 0.02	0.01 ± 0.01	0.03 ± 0.03	0.02 ± 0.01
	Fast	0.04 ± 0.03	0.01 ± 0.01	0.05 ± 0.03	0.02 ± 0.01	0.08 ± 0.05	0.03 ± 0.02
Small	Regular	0.04 ± 0.04	0.02 ± 0.01	0.05 ± 0.04	0.01 ± 0.01	0.06 ± 0.04	0.02 ± 0.01
	Fast	0.04 ± 0.03	0.03 ± 0.03	0.04 ± 0.04	0.03 ± 0.02	0.07 ± 0.06	0.03 ± 0.03

Absolute averaged position difference and standard deviation from the ground truth value in a regular motion pattern (5 s respiratory) and fast motion pattern (3 s respiratory).

### Target volume and similarity index evaluation

**Figure 6:**
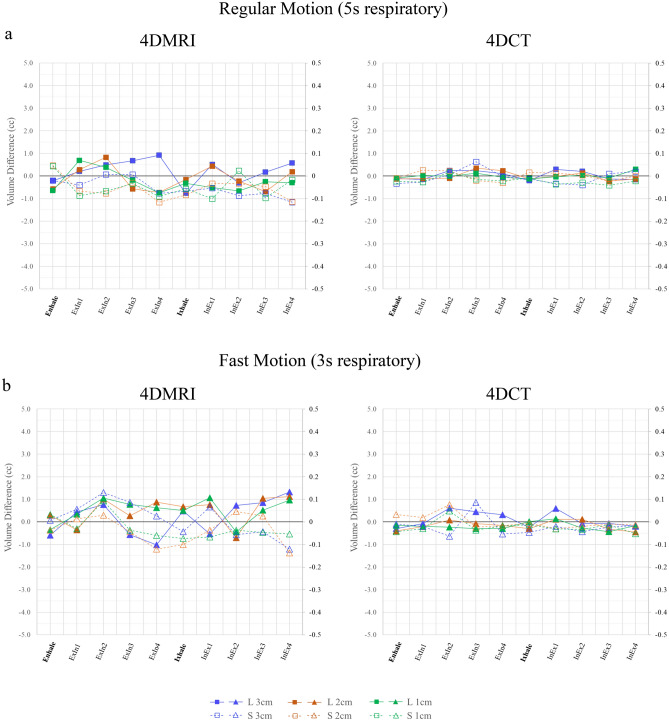
Results of target volume differences for each imaging modality with ten respiratory phases in (**a**) regular motion pattern (5 s respiratory) and (**b**) fast motion pattern (3 s respiratory). The results for the large target (solid marker, left axis) and small target (hollow marker, right axis) are plotted in the same figure. ExIn refers to the phase from exhaling to inhaling, and InEx refers to the phase from inhaling to exhaling. GT refers to ground truth. L and S refer to large and small targets, respectively.

The results of the target volume difference are plotted in Fig. 6. The absolute and percentage volume differences are summarised in Table 2. The volume difference in 4D-MRI and 4D-CT for the regular motion pattern was within 0.48 (3.0%) ± 0.25 cm^3^ and 0.19 (1.3%) ± 0.06 cm^3^ for the large target and 0.06 (11.3%) ± 0.03 cm^3^ and 0.03 (7.6%) ± 0.02 cm^3^ for the small target, respectively. For the fast-motion pattern, the volume difference in 4D-MRI and 4D-CT was observed within 0.72 (4.5%) ± 0.26 cm^3^ and 0.29 (2.1%) ± 0.19 cm^3^ for the large target, and 0.06 (11.2%) ± 0.04 cm^3^ and 0.03 (7.6%) ± 0.02 cm^3^ for the small target, respectively. The Mann–Whitney test showed that 4D-MRI was statistically equivalent to 4D-CT (*p* = 0.13) for large target, but 4D-CT showed improved performance (*p* < 0.01) for small target.

**Table 2:** Absolute averaged volume difference (cc) and percentage difference (%) from the reference.

		Amplitude 1 cm		Amplitude 2 cm		Amplitude 3 cm	
		4DMRI	4DCT	4DMRI	4DCT	4DMRI	4DCT
Large	Regular	0.47 (2.9%) ± 0.20 (1.2%)	0.09 (0.6%) ± 0.08 (0.6%)	0.47 (2.9%) ± 0.23 (1.4%)	0.16 (1.1%) ± 0.09 (0.6%)	0.48 (3.0%) ± 0.25 (1.5%)	0.19 (1.3%) ± 0.06 (0.4%)
	Fast	0.67 (4.1%) ± 0.27 (1.7%)	0.22 (1.5%) ± 0.11 (0.8%)	0.72 (4.4%) ± 0.30 (1.8%)	0.22 (1.6%) ± 0.13 (0.9%)	0.73 (4.5%) ± 0.26 (1.6%)	0.29 (2.1%) ± 0.19 (1.4%)
Small	Regular	0.06 (10.8%) ± 0.03 (5.2%)	0.02 (7.2%) ± 0.01 (2.8%)	0.06 (11.3%) ± 0.03 (5.4%)	0.02 (5.1%) ± 0.01 (2.9%)	0.06 (9.6%) ± 0.03 (6.1%)	0.03 (7.6%) ± 0.02 (5.3%)
	Fast	0.05 (9.3%) ± 0.02 (3.4%)	0.03 (9.6%) ± 0.01 (4.0%)	0.06 (10.4%) ± 0.04 (7.2%)	0.03 (7.6%) ± 0.02 (5.7%)	0.06 (11.2%) ± 0.04 (6.5%)	0.04 (11.6%) ± 0.02 (7.1%)

Absolute averaged volume difference and standard deviation (in cc and percentages) from the reference target volume in a regular motion pattern (5 s respiratory) and fast motion pattern (3 s respiratory).

**Figure 7:**
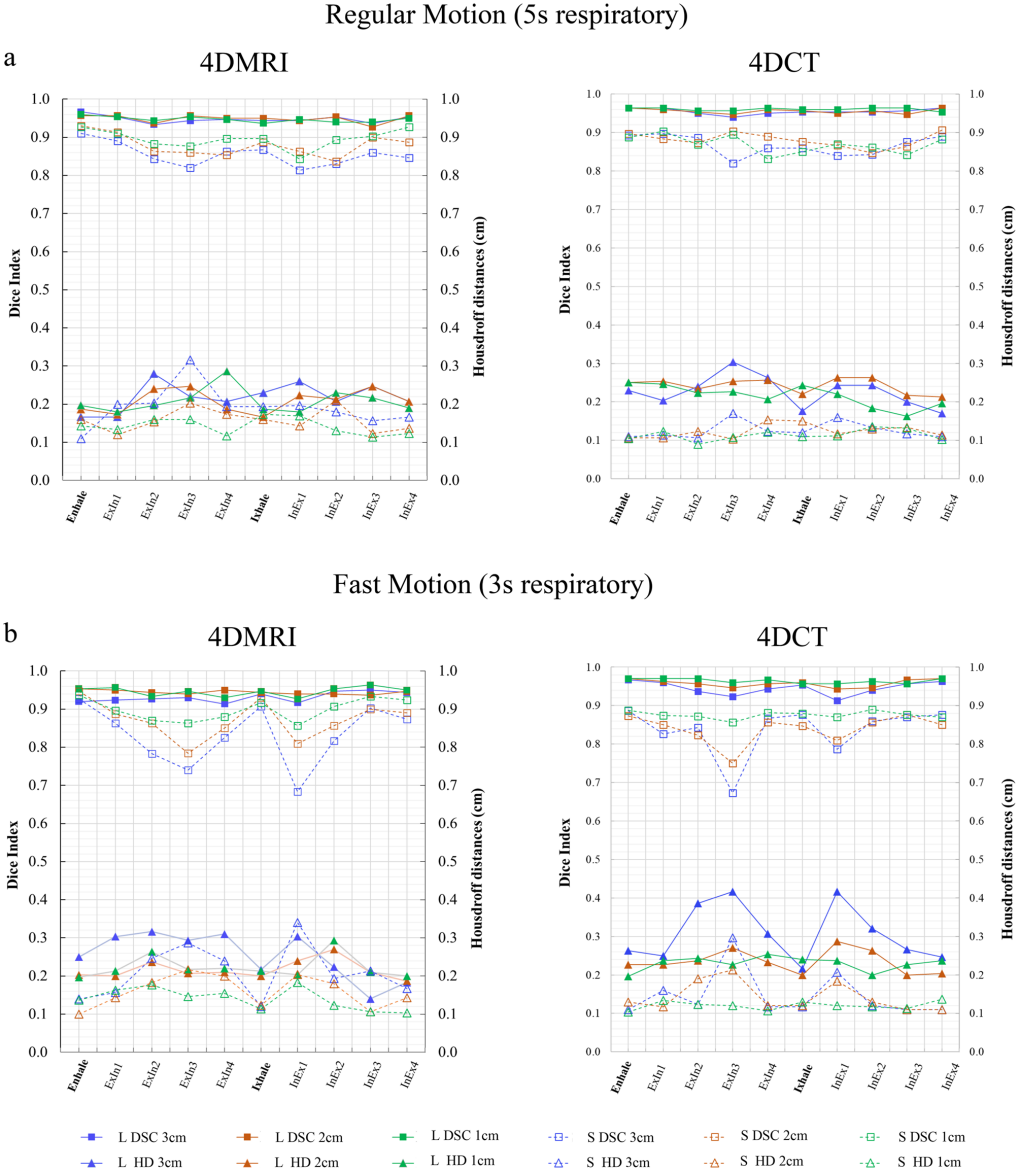
Results of similarity indexes for each imaging modality with ten respiratory phases in (**a**) regular motion pattern (5 s respiratory) and (**b**) fast motion pattern (3 s respiratory). The DSC (solid marker, left axis) and HD (hollow marker, right axis) are plotted in the same figure. DSC refers to Dice index, and HD refers to Hausdorff distance. ExIn refers to the phase from exhaling to inhaling, and InEx refers to the phase from inhaling to exhaling. L and S refer to large and small targets, respectively.

The results of the target similarity index (DSC and HD) analysis for each respiratory phase are plotted in Fig. 7. Small targets with fast-motion patterns were less robust than large targets with regular motion patterns. Phases in ExIn2-3 and InEx1-2, which involved larger movements, exhibited poor similarity index values (shown as low DSC or high HD). For the two similarity indices summarised in Tables 3 and 4, 4D-MRI and 4D-CT showed comparable results. In particular, the minimum average DSC in 4D-MRI and 4D-CT was 0.93 ± 0.01 and 0.95 ± 0.02 for the large target and 0.83 ± 0.07 and 0.84 ± 0.06 for the small target, respectively. Contrastingly, the maximum average HD values in 4D-MRI and 4D-CT were 0.25 ± 0.06 cm and 0.31 ± 0.07 cm for the large target and 0.21 ± 0.06 cm and 0.15 ± 0.06 for the small target, respectively. Although the two modalities have equivalent similarity indices, the results of the Mann–Whitney test showed that 4D-CT has numerically improved performance compared to 4D-MRI; specifically, *p* < 0.01 and* p* = 0.09 for large and small targets in terms of DSC, respectively, and *p* < 0.01 for both targets in terms of HD. Included in Supplementary Material 3 is a detailed statistical summary of our study.

**Table 3:** Averaged Dice Index.

		Amplitude 1 cm		Amplitude 2 cm		Amplitude 3 cm	
		4DMRI	4DCT	4DMRI	4DCT	4DMRI	4DCT
Large	Regular	0.95 ± 0.01	0.96 ± 0.0	0.95 ± 0.01	0.96 ± 0.01	0.95 ± 0.01	0.95 ± 0.01
	Fast	0.95 ± 0.01	0.96 ± 0.01	0.94 ± 0.01	0.96 ± 0.01	0.93 ± 0.01	0.95 ± 0.02
Small	Regular	0.90 ± 0.02	0.87 ± 0.02	0.88 ± 0.03	0.88 ± 0.02	0.85 ± 0.03	0.87 ± 0.02
	Fast	0.90 ± 0.03	0.88 ± 0.01	0.87 ± 0.05	0.84 ± 0.04	0.83 ± 0.07	0.84 ± 0.06

Averaged Dice index and standard deviation in a regular motion pattern (5 s respiratory) and fast motion pattern (3 s respiratory).

**Table 4:** Averaged Hausdorff distance (cm).

		Amplitude 1 cm		Amplitude 2 cm		Amplitude 3 cm	
		4DMRI	4DCT	4DMRI	4DCT	4DMRI	4DCT
Large	Regular	0.21 ± 0.03	0.22 ± 0.03	0.21 ± 0.03	0.24 ± 0.02	0.22 ± 0.03	0.23 ± 0.04
	Fast	0.22 ± 0.03	0.23 ± 0.02	0.22 ± 0.02	0.23 ± 0.03	0.25 ± 0.06	0.31 ± 0.07
Small	Regular	0.14 ± 0.02	0.11 ± 0.01	0.16 ± 0.03	0.12 ± 0.02	0.19 ± 0.05	0.13 ± 0.02
	Fast	0.14 ± 0.03	0.12 ± 0.01	0.16 ± 0.04	0.14 ± 0.04	0.21 ± 0.06	0.15 ± 0.06

Averaged Hausdorff Distance (cm) and standard deviation in a regular motion pattern (5 s respiratory) and fast motion pattern (3 s respiratory).

## Discussion

In this study, we developed a sub-second high temporal-spatial resolution T1-weighted 4D-MRI using a volumetric acquisition scheme with compressed sensing. Additionally, we systematically investigated the 4D-MRI feasibility for target motion assessment using a motion phantom. The 4D-MRI exhibited similar geometrical accuracy compared with 4D-CT in terms of the centroid position, volume, and similarity index. The results suggest that the proposed volumetric T1-weighted 4D-MRI sequence is feasible for accurate assessment of target movements.

The position accuracy results between in 4D-MRI and 4D-CT were comparable to those reported in previous phantom studies. For a 3-cm target, Yue et al.^19^ calculated the error as 0.02 and 0.04 cm for fast and relatively slow motions. In the present study, we calculated the position error as 0.02 and 0.03 cm for fast and regular motions. These results confirmed that a volumetric-scan-based 4D-MRI with compressed sensing could achieve excellent position accuracy comparable to a 2D multi-slice-basis 4D-MRI. For a 1-cm small target that was rarely investigated, the average errors were observed within 0.08 cm in volumetric T1-weighted 4D-MRI, which was larger than those of 4D-CT (within 0.03 cm). This is because of the insufficient temporal resolution and relatively slow 3D-readout process in volumetric 4D-MRI, affecting the accuracy of the centroid position derived from the target ROI. To mitigate the potential influence of resolution discrepancies between two modalities, we resampled images for both modalities to a resolution of 1 × 1 × 1 mm^3^ prior to performing ROI generation. Furthermore, our analysis of position accuracy revealed that the displacement of the target ROI for both modalities was within 1 mm, which is deemed acceptable since it falls below the 1-pixel resolution used for ROI generation. The results of the volume difference and similarity index indicate that the volumetric T1-weighted 4D-MRI and 4D-CT can achieve similar accuracies. In particular, the similarity index was applied in our study because the target volume might underestimate the motion-induced shape change (e.g., same volume but different shape). The results show that the volume difference is usually correlated with DSC and HD, compared to the DSC, providing more direct insights into the motion-induced artefacts. In DSC for small targets in the proposed 4D-MRI, phases ExIn3 and InEx1 were observed to be most vulnerable to the motion artefacts along the motion direction. The insufficient temporal resolution and relatively slow process for 3D-readout in the proposed sequence might cause the artefacts to appear as a shape deformation along the movement direction. In contrast, the movement trajectory was also captured in the volumetric-based MR scan, suggesting that 4D-MRI has no limitations in evaluating the target movement range and defining internal target volume.

The literature contains several studies discussing the use of 4D-MRI utilizing 3D sequences. Feng et al.^23^ developed a framework called iGRASP for image reconstruction, which enhances image quality and reduces motion-blurring artifacts in liver dynamic contrast-enhanced MRI. Oar et al.^17^ employed the stack-of-stars radial trajectory^24^, which acquires data through the center of k-space, to generate respiratory phase-resolved 4D-MRI with reduced motion artifacts. Although, these studies did not provide details on the temporal resolution, their temporal resolution was limited to more than 10 s^23^. To achieve sub-second high temporal resolution, our developed sequence features a single-shot 3D acquisition technique, which incorporates both half-scan and compressed sensing techniques. Despite its merits, the approach presents potential impediments, including a decrease in contrast due to an elevated TFE factor and a limited slice availability (between 2.4 and 4.5 cm in the current study). Therefore, we utilized an enhanced hepatobiliary phase T1-weighted MRI, ensuring high-temporal-resolution imaging without compromising image contrast. The 3D acquisition was performed in the sagittal plane, covering a FOV size of 256 × 256 mm^2^ to capture the mostly respiratory-induced motion direction (superior-inferior(S-I) and anterior–posterior(A-P) direction). Though respiratory motion typically exerts less influence on the left–right(L-R) direction, the available slice range could impact assessments of movements exceeding 7 mm in the L-R direction under the current setting. Nevertheless, their findings suggest a strong potential for combining the motion-robustness of radial imaging with compressed sensing techniques, which could be integrated into our sequence. Biederer et al.^25^ utilized an artificial chest phantom to investigate the diaphragm dome displacements between 4D-MRI and 4D-CT. Despite the significant spatial resolution difference between the two modalities in their study, the authors emphasized the importance of temporal resolution in evaluating 4D-MRI. We concur with this notion, and it served as the driving force for our current work. Besides, our findings showed that 4DCT exhibits better numerical accuracy compared to 4DMRI, such as with a target ROI displacement of 0.03 cm in 4D-CT and 0.08 cm in 4D-MRI for small targets. However, it is important to note that both modalities show sub-millimeter ROI displacement, which falls below the resolution used for ROI generation and is considered comparable. Similar results were observed when comparing the volume differences and similarity indices between the two modalities. Therefore, the conclusion of our study is not to assert the superiority of 4DMRI over 4DCT but to state that 4DMRI, in its current stage, can achieve a level of accuracy that is comparable to 4DCT. Further, Yuan et al.^18^ demonstrated a rapid 4D MR acquisition using a 3D scheme, achieving a sub-second to 0.6-s temporal resolution. The reported spatial resolution (2.7 × 2.7 × 4 mm^3^) might limit the accuracy of evaluation for small targets (e.g., 1 cm in diameter), as only 2–3 slices may be available for target delineation in a 4 mm out-of-plane resolution. It's generally recognized that an enhancement in spatial resolution might correspondingly deteriorate image quality. However, considering that the principal objective of employing 4D-MRI in radiotherapy is to assess the motion of the target, rather than mapping the entire abdominal area, our approach strategically confines the FOV and slice range to a suitable degree, without hindering the evaluation of motion in the most significant directions. Through this optimized methodology, we've managed to achieve a 0.5-s temporal resolution, which exceeds the performance of previous studies while simultaneously maintaining high spatial resolution.

In terms of clinical implications, the findings of our study have several potential applications. Increasingly, the evidence-based advantages of radiotherapy, notably SBRT, in liver cancer treatment are acknowledged, providing exceptional local control, improved survival rates, and minimal adverse effects^26,27^.Notably, tumors smaller than 5 cm have been shown to have particularly favorable outcomes^26,28^. Our proposed methodology enhances this approach by offering a more intricate and comprehensive evaluation of tumor motion. Moreover, it holds significant potential for managing smaller tumors (e.g., 1 cm in size), a task that presents substantial challenges to current techniques. The proposed method could assist in the precision of target margin setting and treatment planning, resulting in a potential enhancement of the therapy's effectiveness and a reduction in its side effects. Nevertheless, the current method has a relatively restricted scan scope in out-of-plane could limit the assessment of larger tumors under high spatial resolution conditions. Concurrently, the potential usability of MRI-linac using 4D MRI has also been highlighted, with improved target coverage and reduced dose to organs at risk compared to conventional radiotherapy^29^. Despite uncertainties related to dosimetric impact of the contrast agent, our approach offers a promising imaging guide sequence for future 4D-MRI guided online adaptation by MRI-linac. Moreover, many hepatocellular carcinoma patients have chronic liver diseases, posing potential risks of liver decompensation post-SBRT^30^. Recent suggestions^31^ propose individualized adaptive SBRT using the indocyanine green retention test as a liver function marker. Contrastingly, our method could provide a non-invasive technique for evaluating both overall and local liver functionality, extending its utility beyond merely a motion management strategy^13,32^.

The present study has a few limitations that need to be addressed in future investigations. First, given the experiment's magnitude, we had to restrict our study to a single-axis (in S-I direction) sigmoidal motion, which typically represent the most prominent direction of movements. It's noteworthy that in-plane resolution (S-I and A-P direction) surpasses that of out-of-plane (L-R direction), suggesting that motion occurring within the in-plane directions is likely to be measured with a higher degree of accuracy. Multi-axis movement, including a higher proportion of out-of-plane motion, might detrimentally affect the precision of positioning and volume estimation. Further investigations are required to evaluate the impacts of multi-axis motion, which is frequently encountered in real patient scenarios. In addition, surrogate signals using different approaches were not investigated. Prior studies have shown that internal navigator or self-navigation techniques might have fewer binning motion artefacts and more robustness against irregular breaths^33,34^. However, Stemkens et al.^7^ argued that the self-navigation method has limitations, including phase instability and partial saturation bands, particularly in the case of 2D image-based self-navigators. It is vital to understand that in a phantom study, surrogate indicators, whether external or internal, have a minimal impact on the outcome due to the rigid and consistent nature of phantom motion. Although exploring various surrogate signals is beyond the scope of current phantom study, we wish to underline that, in actual patients, the external signal might differ from the external signal. Moreover, the implementation of a respiratory phase sorting/reconstruction algorithm is expected to deal with irregular breathing patterns and better simulate real patient cases^17,35^. Unfortunately, we are currently unable to incorporate a sorting/reconstruction algorithm into our sequence since the techniques are still in development. Therefore, in this study, we utilized a respiratory gating sensor to acquire 10-phase MR images, which were binned in a sequential manner based on the respiratory profile. A comparable approach was also reported in a previous study^18^. While our study focused on achieving high temporal resolution in 4DMRI acquisition, we acknowledge the need for a respiratory phase sorting/ reconstruction algorithm to enhance its clinical utility. High temporal-spatial resolution MRI with satisfactory target-to-background contrast was verified using the introduced scanning parameters. However, scanning a patient with large body sizes or more dynamic tissue densities might be challenging. Further investigation is required to ensure whether the proposed 4D-MRI could provide clinically acceptable image quality for real patients. Moreover, Further improvements in temporal-spatial resolution and image quality in 4D-MRI might not necessarily be limited by hardware development or image acquisition techniques. The trade-off between escalating temporal-spatial resolution and preserving image quality could be significantly optimized by employing artificial intelligence-enhanced image processing techniques^36–38^. A recent study^36^ highlights a deep learning-driven super-resolution method for MRI, achieving 2-3x (potentially up to 4x) enhanced resolution while maintaining image quality. This indicates the feasibility of achieving sub-millimeter spatial resolution in both in-plane and out-of-plane directions with our proposed method. Moreover, it suggests an alternative approach to expanding the scan scope in the out-of-plane direction of our proposed sequence by adjusting the scan resolution (e.g., 5 mm) for larger targets, while maintaining a final output at 2-mm spatial resolution. In addition, a variety of deep learning-oriented techniques for noise and artifact reduction in MRI have been proposed, tested on diverse datasets, and demonstrated substantial enhancements in image quality^37^. This suggests the potential to further increase the temporal resolution without compromising image contrast. Further, study from Liu et al*.*^38^ extends the advantages of using 4D MRI over 4DCT, not only for motion assessment but also for dose calculation. The creation of synthetic 4DCT based on 4DMRI enables direct dose calculation for treatment planning and also shows potential for online adaptation with MRI-linac.

In conclusion, while 4D-CT may demonstrate superior numerical accuracy in phantom studies, the proposed high temporal resolution 4D-MRI demonstrates sub-millimetre geometric accuracy comparable to that of 4D-CT. These findings suggest that the 4D-MRI technique is a viable option for characterizing motion and generating phase-dependent internal target volumes within the realm of radiotherapy.

### Supplementary Information

Supplementary Information 1.

Supplementary Information 2.

Supplementary Information 3.

## Data Availability

The datasets used and/or analysed during the current study available from the corresponding author on reasonable request.

## Abbreviations

4D-CT: Four-dimensional computed tomography
4D-MRI: Four-dimensional magnetic resonance imaging
DSC: Dice similarity coefficient
HD: Hausdorff distance
GT: Ground truth
V_measured_: Measured volume
V_ref_: Reference volume
ExIn: Respiratory phase from exhaling to inhaling
InEx: Respiratory phase from inhaling to exhaling
SBRT: Stereotactic body radiation therapy
