# Supplementary material for: Comparative study of sub-second temporal resolution 4D-MRI and 4D-CT for target motion assessment in a phantom model
Source: Sci Rep. 2023 Sep 21;13:15685. doi: 10.1038/s41598-023-42773-z (PMC10514030; doi:10.1038/s41598-023-42773-z)
Supplement: Supplementary file 1 — Supplementary Information 1. [file 41598_2023_42773_MOESM1_ESM.pdf]

Supplementary material 1

a

Large Target (5s / 2cm)

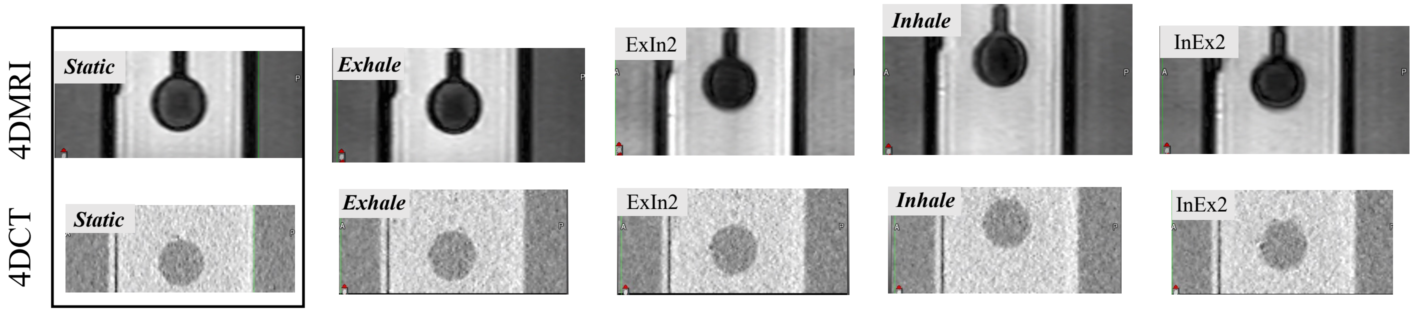

Large Target (3s / 3cm)

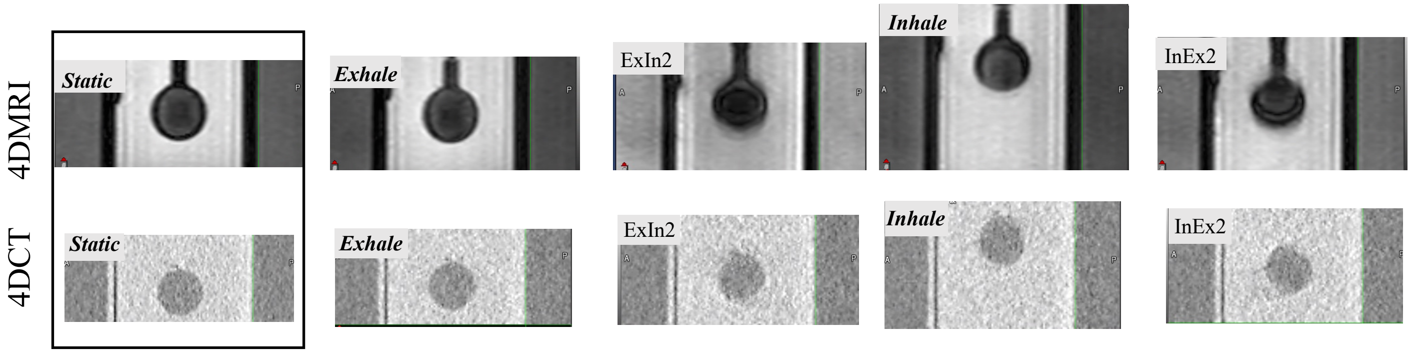

b

Small Target (5s / 2cm)

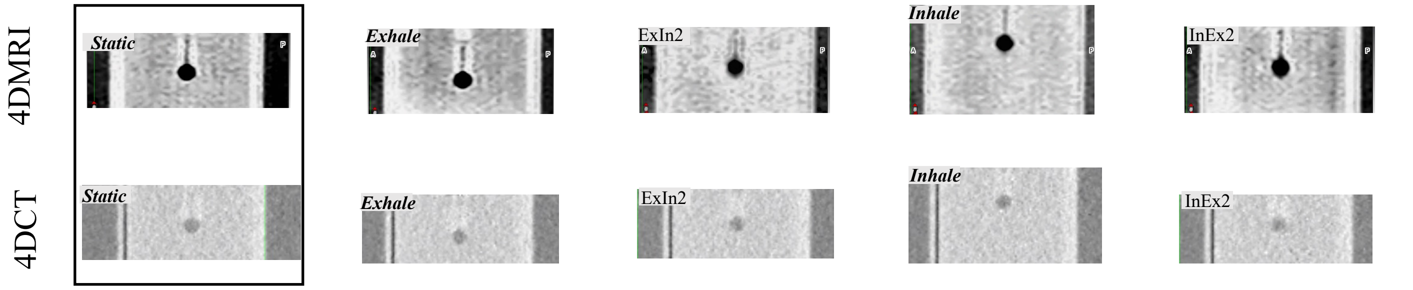

Small Target (3s / 3cm)

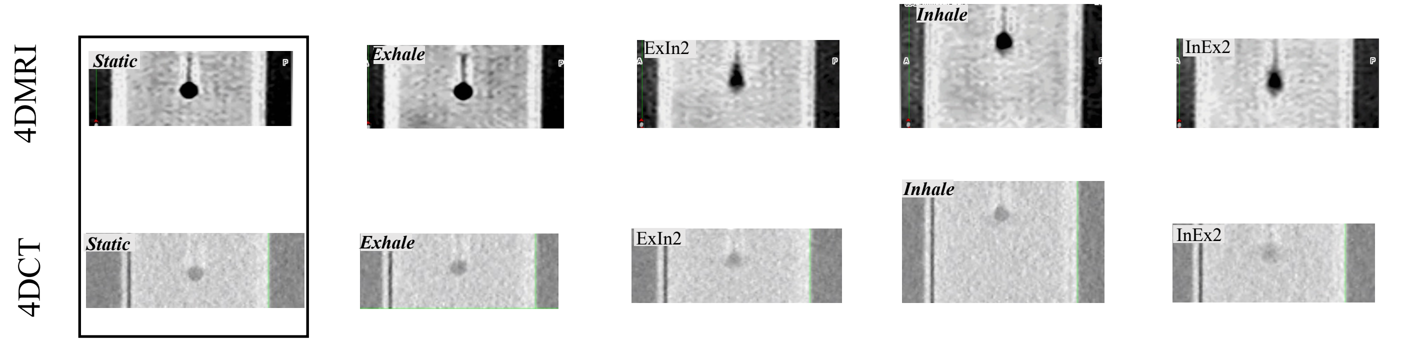

Supplementary material 1:

A zoomed-out view of the 4D-MRI and 4D-CT images with four respiratory phases in regular (5 s/2 cm) and fast (3 s/3 cm) motion patterns. The upper and lower panels show the results of large and small targets, respectively. The static image is shown on the left as a reference. ExIn refers to the phase from exhaling to inhaling, and InEx refers to the phase from inhaling to exhaling.
