# Supplementary material for: Comparative study of sub-second temporal resolution 4D-MRI and 4D-CT for target motion assessment in a phantom model
Source: Sci Rep. 2023 Sep 21;13:15685. doi: 10.1038/s41598-023-42773-z (PMC10514030; doi:10.1038/s41598-023-42773-z)
Supplement: Supplementary file 2 — Supplementary Information 2. [file 41598_2023_42773_MOESM2_ESM.pdf]

Supplementary material 2

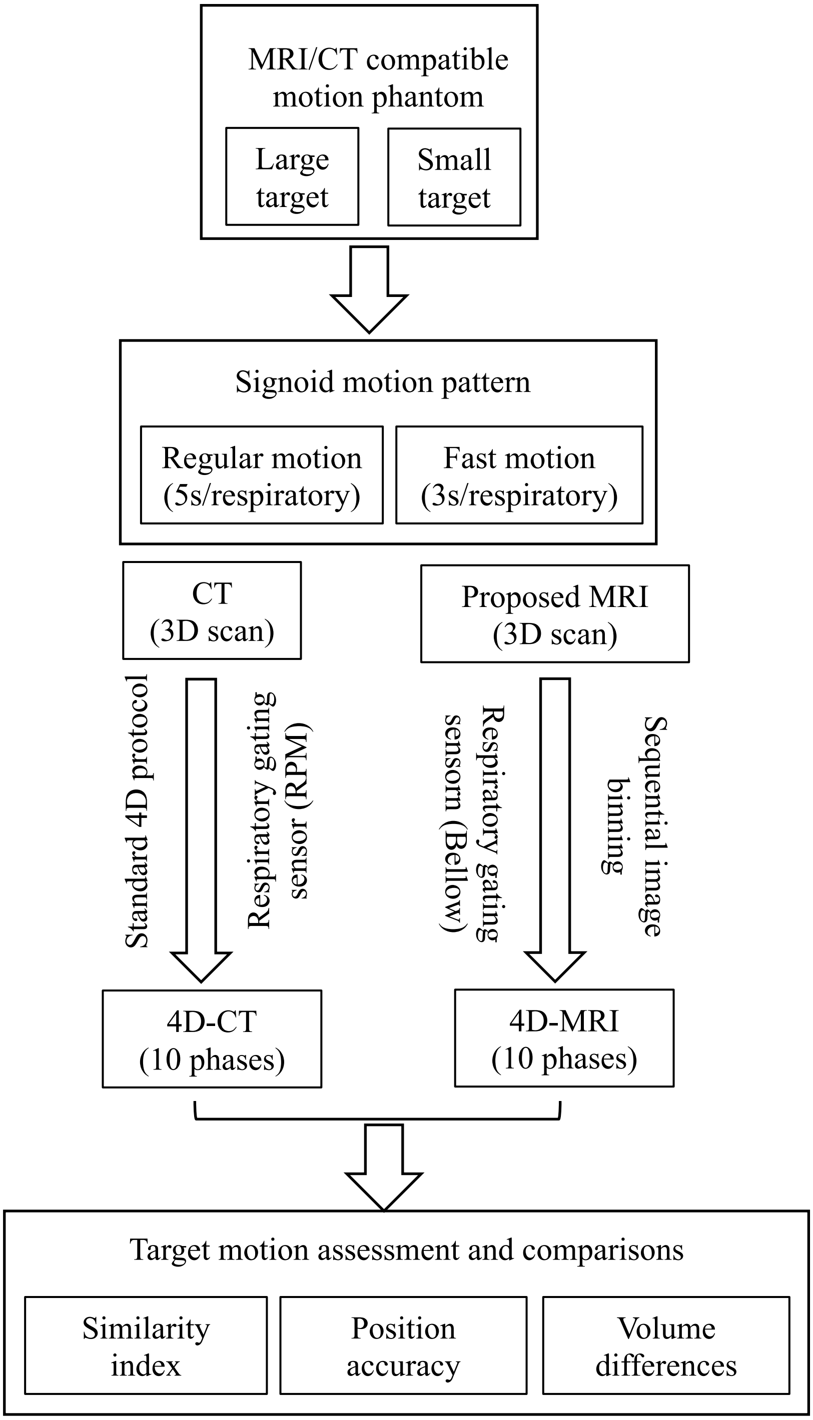

Supplementary material 2:

A flowchart summarizing the outline of the methodology employed in this study.
