# Supplementary material for: Comparative study of sub-second temporal resolution 4D-MRI and 4D-CT for target motion assessment in a phantom model
Source: Sci Rep. 2023 Sep 21;13:15685. doi: 10.1038/s41598-023-42773-z (PMC10514030; doi:10.1038/s41598-023-42773-z)
Supplement: Supplementary file 3 — Supplementary Information 3. [file 41598_2023_42773_MOESM3_ESM.pdf]

Supplementary material 3

	4DMRI vs 4DCT for large target			4DMRI vs 4DCT for small target		
	Total (n=60)	Regular motion (n=30)	Fast motion (n=30)	Total (n=60)	Regular motion (n=30)	Fast motion (n=30)
Position Diff	*< 0.01*	*0.02*	*< 0.01*	*< 0.01*	*< 0.01*	0.07
Volume Diff	0.10	0.25	*0.01*	*< 0.01*	*< 0.01*	0.38
DI	*< 0.01*	*< 0.01*	*< 0.01*	0.09	0.56	0.09
HD	*< 0.01*	*0.04*	0.06	*< 0.01*	*< 0.01*	*< 0.01*

Supplementary material 3:

Statistical Summary of the Study. The results are derived from the Mann-Whitney U-test with a two-tailed p-value. Significance was established at $p<0.05$, with underlined p-values indicating non-significant results. DI refers to Dice index, and HD refers to Hausdorff distance. A regular motion pattern refers 5 s/2 cm, and a fast motion pattern refers to 3 s/3 cm.
